# Study of Microbiomes in Aseptically Collected Samples of Human Breast Tissue Using Needle Biopsy and the Potential Role of *in situ* Tissue Microbiomes for Promoting Malignancy

**DOI:** 10.3389/fonc.2018.00318

**Published:** 2018-08-17

**Authors:** Shen Meng, Bin Chen, Junjie Yang, Jingwen Wang, Dequan Zhu, Qingsong Meng, Lei Zhang

**Affiliations:** ^1^School of Medicine and Life Sciences, Shandong Academy of Medical Sciences, University of Jinan, Jinan, China; ^2^Shandong Cancer Hospital Affiliated to Shandong University, Jinan, China; ^3^College of Life Science, Shandong Normal University, Jinan, China; ^4^College of Life Science, Qilu Normal University, Jinan, China; ^5^Microbiological Laboratory, Lin Yi People's Hospital, Linyi, China; ^6^Clinical Laboratory, Qianfoshan Hospital Affiliated to Shandong University, Jinan, China; ^7^Shandong Children's Microbiome Center, Qilu Children's Hospital of Shandong University, Jinan, China; ^8^Shandong Institutes for Food and Drug Control, Jinan, China; ^9^Qingdao Human Microbiome Center, No. 2 Affiliated Hospital of Qingdao University, Qingdao, China; ^10^Beijing Advanced Innovation Center for Big Data-Based Precision Medicine, School of Chemistry and Environment, Beihang University, Beijing, China

**Keywords:** microbiome, breast cancer, needle biopsy, malignancy, pathogen

## Abstract

Mounting evidence suggests that changes in microbiome are linked to development of cancer and its aggressiveness. Microbiome profiles in human breast tissue previously presumed to be sterile, have recently been characterized using high-throughput technologies. Recent findings of microbiome variation between benign and malignant disease provides a rationale for exploring microbiomes associated with cancer during tumor progression. We assessed microbiomes of aseptically collected human breast tissue samples in this study, using needle biopsy from patients with benign and malignant tumors of different histological grading, using 16S rRNA gene amplicon sequencing. This is consistent with previous studies, and our results identified distinct microbiome profiles in breast tissues from women with cancer as compared to women with benign breast disease in Chinese cohorts. The enriched microbial biomarkers in malignant tissue included genus *Propionicimonas* and families Micrococcaceae, Caulobacteraceae, Rhodobacteraceae, Nocardioidaceae, Methylobacteriaceae, which appeared to be ethno-specific. Further, we compared microbiome profiles in malignant tissues of three different histological grades. The relative abundance of family Bacteroidaceae decreased and that of genus *Agrococcus* increased with the development of malignancy. KEGG pathways inferred by PICRUSt showed that biotin and glycerophospholipid metabolism had significant differences in all three grades. Glycerophospholipid and ribosome biogenesis increased in grade III tissue as compared to grades I and II. Flavonoid biosynthesis significantly decreased in grade III tissue. The specific correlation of these potential microbial biomarkers and indicated pathways with advanced disease could have broad implications in the diagnosis and staging of breast cancer. Further large-cohort investigation of the breast cancer microbiome and its potential mechanism in breast cancer development are essential.

## Introduction

The recent appreciation of the influence of microbiota on human health and disease sheds light on whether microbes play a role in cancer development and progression (1). While several studies on microbes and their relationship to cancer have focused on the gut microflora (2), growing awareness of the presence of microbes within and adjacent to the tumor site has also led to a host of findings, which is important for unveiling mechanisms of microbiota and associated microenvironment in carcinogenesis (3). Microbiomes have been implicated in cancer development and progression in specific body sites including stomach (4), colon (5), liver (6), lungs (7), and skin (8). It has become apparent that both community composition and discrete bacterial species can exert either pathogenic effects that encourage disease development or probiotic effects that maintain health status. Although breast cancer has been among the earliest and most intensely-studied diseases using genomic technology (9), it is not until recently that the existence of microbes in breast tissue and the potential role of breast ductal microbiome *in situ* were explored (10, 11). The breast consists of epithelium, stroma and a mucosal immune system that make up a complex microenvironment (12). Since mucosal immune systems develop as a direct result of microbial exposure and inflammation is associated with the promotion of various malignancies, partly due to bacterial infection-induced microenvironmental changes, the presence of immune effectors within the complex microenvironment of the breast is suggestive of a breast microbiome (13, 14). More recently, the differences in the microbiome of human breast tissue from women with benign and malignant disease provided insights for subsequent investigations on the role of the breast microbiome in breast carcinogenesis and breast cancer prevention (15). However, variations in microbiome profiles between different histological grades of breast malignancy have not been evaluated. In this study, we characterized and compared the microbiome of aseptically collected human breast samples from patients with benign and malignant cancer having different histological grades using needle biopsy and 16S rRNA gene amplicon sequencing. Phylogenetic Investigation of Communities by Reconstruction of Unobserved States (PICRUSt) was used to infer KEGG pathways in microbiomes of benign and different malignant tumors.

## Materials and methods

### Patients and sample procurement

This study was approved by the Institutional Review Board of Qianfoshan Hospital affiliated to Shandong University. We enrolled 94 patients undergoing non-mastectomy breast surgeries in our study and obtained written informed consent from all patients (Table S1). Research was performed in accordance with relevant guidelines and regulations and patients who were pregnant or lactating were excluded, and patients receiving antibiotics within 6 months were not eligible, and neither were patients with any other disease or condition that might interfere with the study assessments. Breast tissue was collected using aseptic percutaneous needle biopsy. After surgery, samples were immediately placed in sterile tubes, stored at −196°C in a nitrogen canister and then transferred to a −80°C freezer until processing.

### DNA extraction and 16S rRNA gene sequence

DNA extraction was performed with a DNeasy Blood & Tissue Kit (Qiagen) according to the manufacturer's instruction. Quantitation of DNA was measured using NanoDrop 2000 (Thermo Scientific).

To generate 16S rRNA gene amplicons, in a 50 ul reaction, typically 50 ng of DNA was used as a template, with 0.4 uM of V1-V2 barcoded primers targeting 27F and 355R of the bacterial 16S rRNA gene (5′ AGAGTTTGATCMTGGCTCAG3′ and 5′ GCTGCCTCCCGTAGGAGT3′). Purified with QIAquick PCR Purification Kit and (Qiagen) PCR purification procedure, all amplicons were quantified and then pooled to equalize concentrations for sequencing, using HiSeq 2500 (Illumina).

### 16S rRNA gene sequence analysis

The 16S rRNA gene sequence paired-end data set was joined and quality filtered using the FLASH method, described by Magoč and Salzberg (16). Sequencing analysis was conducted in the Quantitative Insights Into Microbial Ecology (QIIME, version 1.9.1) software suite (17), according to the QIIME tutorial (http://qiime.org/) with some modifications. Chimeric sequences were removed using usearch61 (18) with *de novo* models. Sequences were clustered against the Greengenes (13_8 release) ribosomal database's 97% reference data set. Sequences that did not match any entries with this reference were subsequently clustered into *de novo* OTUs at 97% similarity with UCLUST. Taxonomy was assigned to all OTUs using the RDP classifier (19) within QIIME and the Greengenes reference data set. Rarefaction and rank abundance curves were calculated from OTU tables using alpha diversity and rank abundance scripts within the QIIME pipeline. The hierarchical clustering based on population profiles of most common and abundant taxa was performed using UPGMA clustering (Unweighted Pair Group Method with Arithmetic mean, also known as average linkage) on the distance matrix of OTU abundance. This resulted in a newick format tree, which was obtained utilizing the QIIME package.

### Statistical analysis

To account for any bias caused by uneven sequencing depth, the least number of sequences present in any given sample from a sample category was selected randomly prior to calculating community-wide dissimilarity measures (α-diversity and β-diversity). We then rarefied the OTU table to a sequencing depth of 25,000 per sample for both diversity analyses. All Principal Coordinate Analyses (PCoA) were based on unweighted and weighted UniFrac distances using even OTU samples. The prediction of the functional composition of a metagenome using marker gene data and a database of reference genomes was done with PICRUSt as described by Langille et al. (20). The graphical representation of the results was done with STAMP (21) and the calculation of *P*-values was done with Kruskal-Wallis *H*-test and Welch's *t*-test.

## Results

### Breast tissue microbiome in benign vs. malignant disease

**Figure 1:**
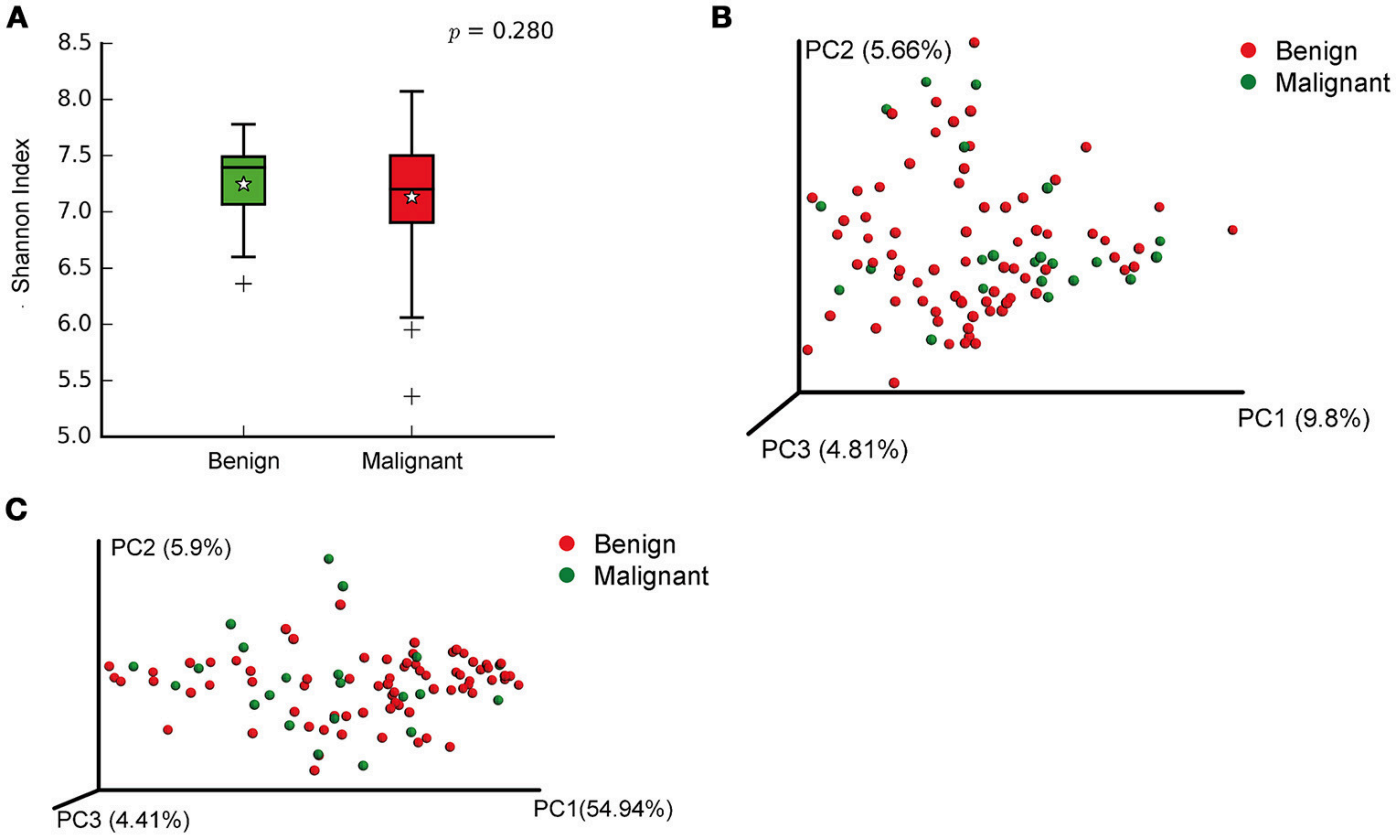
Alpha and Beta diversity in benign and malignant breast tissue. **(A)** Boxplot compares Shannon index between benign and malignant (*P* = 0.280, Kruskal-wilcoxs *H*-test); **(B)** PCoA plots show the clustering pattern of the two groups based on unweighted UniFrac distance (*P* = 0.342, Adonis); **(C)** PCoA plots show the clustering pattern of the two groups based on weighted UniFrac distance (*P* = 0.858, Adonis).

**Figure 2:**
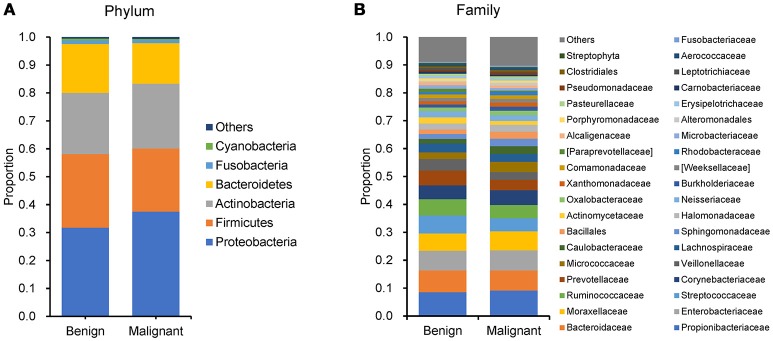
Barplots of the taxonomic profiles of the benign and malignant breast tumor tissue microbiota. (**A)** Phylum level; **(B)** Family level.

**Figure 3:**
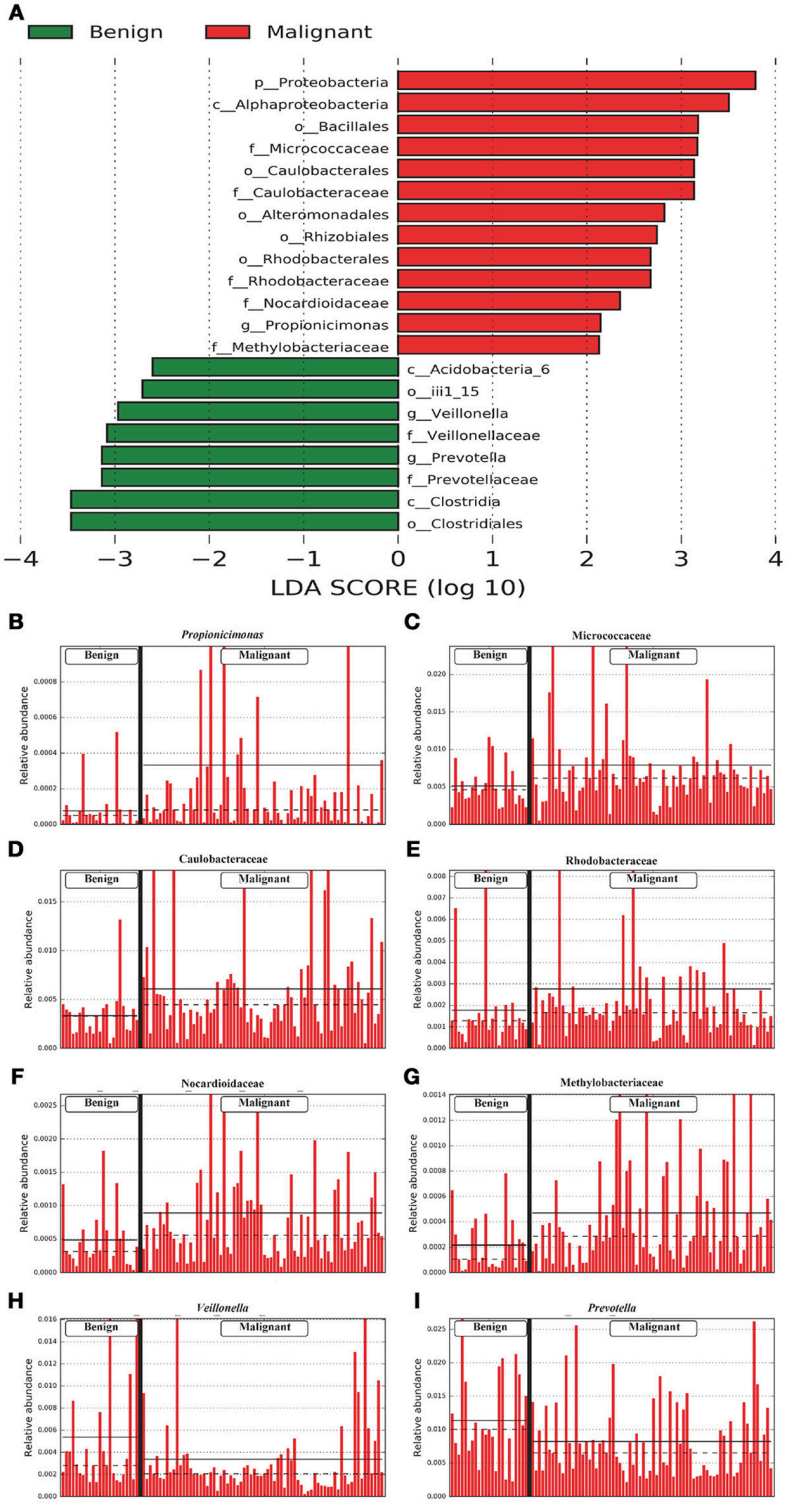
LefSe identified the most differential microbiota between benign and malignant tumors. **(A)** Histogram of the LDA scores computed for abundance between benign and malignant. Benign are indicated with a negative LDA score, and malignant had a positive score. The LDA score also indicates the effective size and ranking of each differentially abundant taxon. **(B–I)**. Barplots show the abundances of the three differential genera **(B,H,I)** and five differential families **(C–G)** between benign and malignant. Each bar represents a sample.

We compared the microbiome profiles in 22 benign and 72 malignant breast cancer patients (Table 1). Alpha diversity analysis reveals that there was no significant difference in Shannon index between benign and malignant disease states (*P* = 0.280, Kruskal-Wilcox's *H*-test, Figure 1A). The benign disease had a slightly higher alpha diversity. To visualize the overall differences in beta diversity between the microbiome profiles of two groups, we conducted Principal Coordinate Analysis (PCoA) of weighted and unweighted UniFrac distances. Although the generated weighted and unweighted UniFrac distance metric did not show significant differences in beta diversity between benign and malignant disease states (Figures 1B,C), the microbial composition of benign and malignant disease states did differ at the phylum and family levels (Figures 2A,B). At the phylum level, Proteobacteria accounted for the major bulk of bacteria (31.77% vs. 37.55%), Firmicutes (26.36% vs. 22.56%), Actinobacteria (21.9% vs. 23.2%) and Bacteroidetes (17.53% vs. 14.57%). The relative abundance of Proteobacteria in malignant disease is significantly higher than in benign disease (*P* = 0.0027, Kruskal-Wilcox's *H*-test). To further evaluate microbiome differences between benign and malignant disease, LEfSe analysis was used to discover different compositions of microbiota and identify significant biomarkers (Figure 3A). Genus *Propionicimonas* and five families Micrococcaceae, Caulobacteraceae, Rhodobacteraceae, Nocardioidaceae and Methylobacteriaceae were abundant in malignant disease compared to benign disease, and only two genera were enriched in benign disease state (LDA>2). The histogram shows differences in the quantity of these genera and families (Figures 3B–I). We analyzed the effects of several biological/confounding factors on 21 discovered biomarkers (Table S2). There were 11 out of 21 biomarkers affected by either age or menopausal status (*p* < 0.05), while in the malignant group, only 3 out of 21 biomarkers were affected by either age or CerB02 (*p* < 0.05).

**Table 1:** Demographic information of all subjects used in this study.

**Variable**	**Total (*N* = 94)**	**Benign (*n* = 22)**	**Malignant (*n* = 72)**
**AGE, YEARS**			
Average (range)	52 (29–77)	47 (32–60)	54 (29–77)
**GRADE**			
I	NA	NA	7 (9.7%)
II	NA	NA	36 (50%)
III	NA	NA	13 (18.1%)
NO-grade	NA	NA	16 (22.2%)
**MENOPAUSAL STATUS**			
Pre-menopause	41	16	25
Peri-menopause	3	1	2
Post-menopause	50	5	45
**ER**			
+	NA	NA	47
–	NA	NA	25
**PR**			
+	NA	NA	42
–			30
**CerB-2**			
+	NA	NA	53
–	NA	NA	19

**Figure 4:**
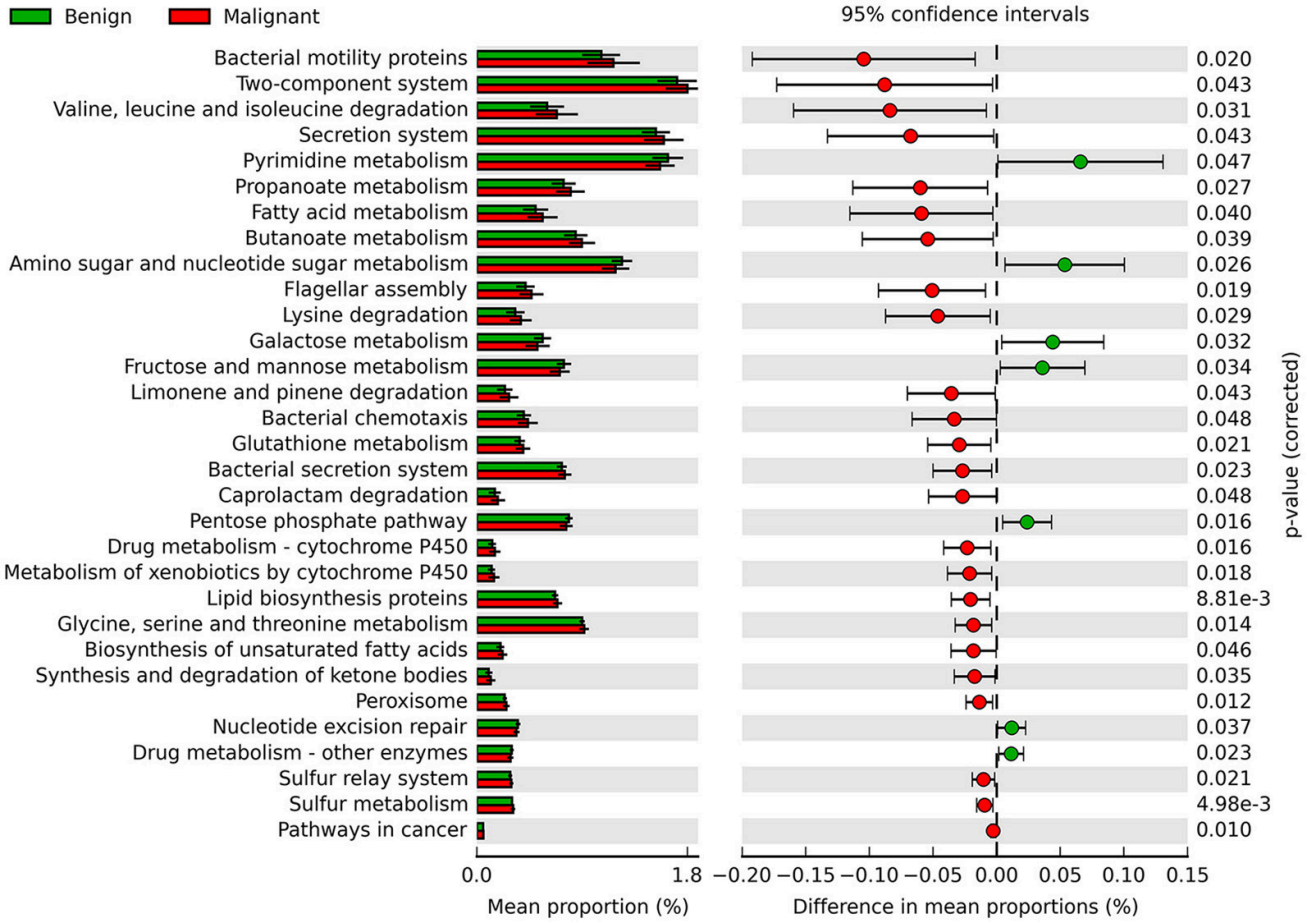
Extended error bar plot showing the significantly different KEGG pathways between benign and malignant tumors. The malignant ones have a positive difference between relative abundances and benign have a negative difference between relative abundances.

Phylogenetic Investigation of Communities by Reconstruction of Unobserved States (PICRUSt) was used to infer the KEGG pathways between the microbiome of benign and malignant states, which revealed 31 different KEGG pathways (Figure 4). Benign tissue showed increased pathways in cancer, lipid biosynthesis proteins, peroxisome, glycine, serine, and threonine metabolism, drug metabolism—cytochrome P450, metabolism of xenobiotics by cytochrome P450 and glutathione metabolism, whereas microbiota in malignant tissue showed reduced amino sugar and nucleotide sugar metabolism and drug metabolism—other enzymes.

### Breast tissue microbiome in different histological grade

**Figure 5:**
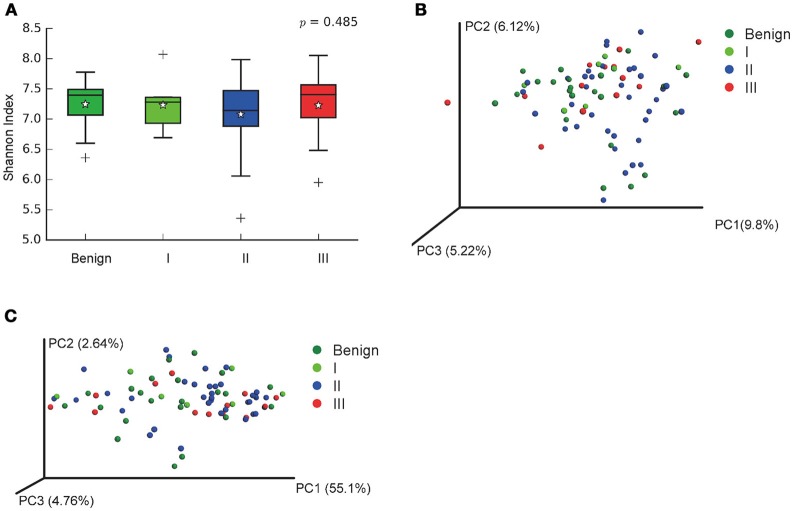
Alpha and Beta diversity in malignant breast tumor tissue of different histological grade. **(A)** Boxplot compares Shannon index between benign and malignant (*P* = 0.540, Kruskal-Wilcox's *H*-test); **(B)** PCoA plots show the clustering pattern of the two groups based on unweighted UniFrac distance (*P* = 0.502, Adonis); **(C)** PCoA plots show the clustering pattern of the two groups based on weighted UniFrac distance (*P* = 0.853, Adonis). ^*^indicates the mean of the data, the data points outside of the whiskers are shown as crosses +.

**Figure 6:**
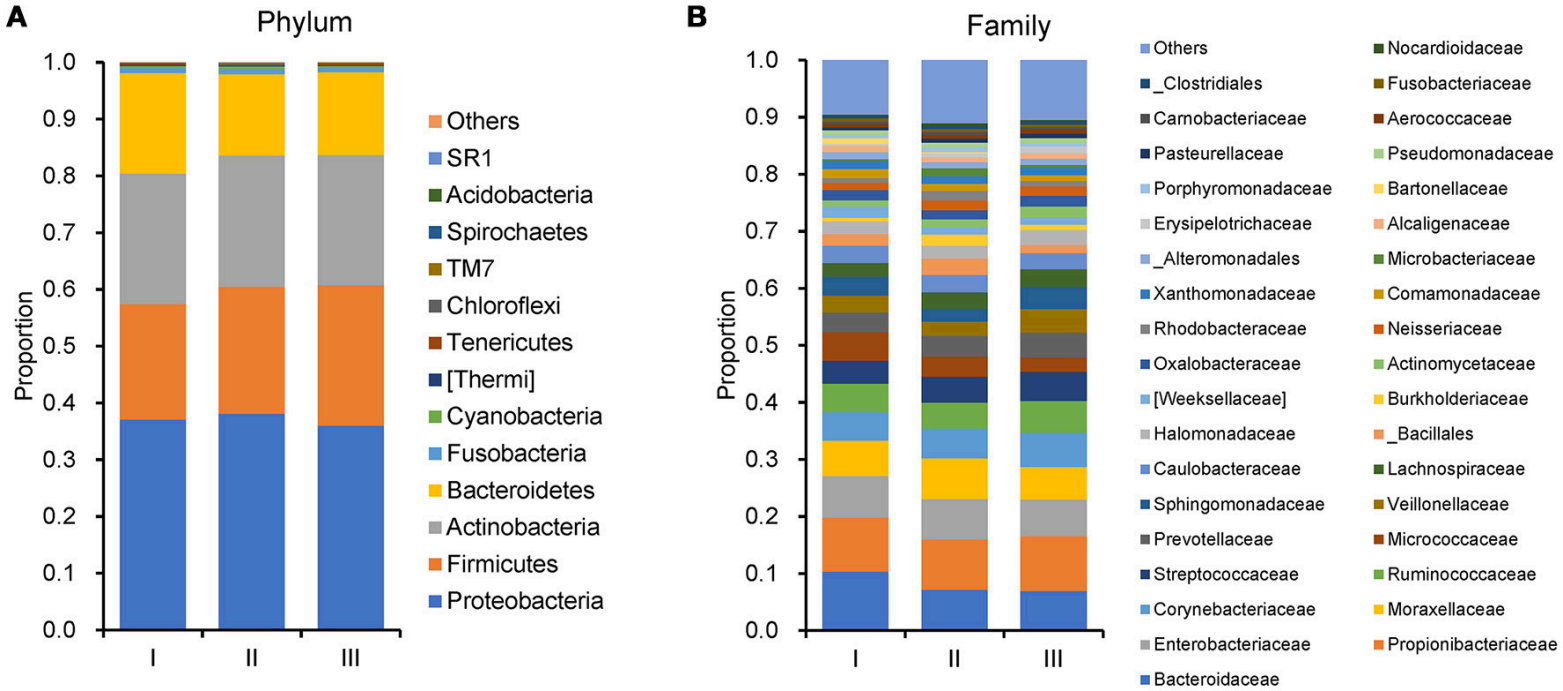
Barplots of the taxonomic profiles of the grade I, II and III breast tumor tissue microbiota. **(A)** Phylum level. **(B)**Family level.

**Figure 7:**
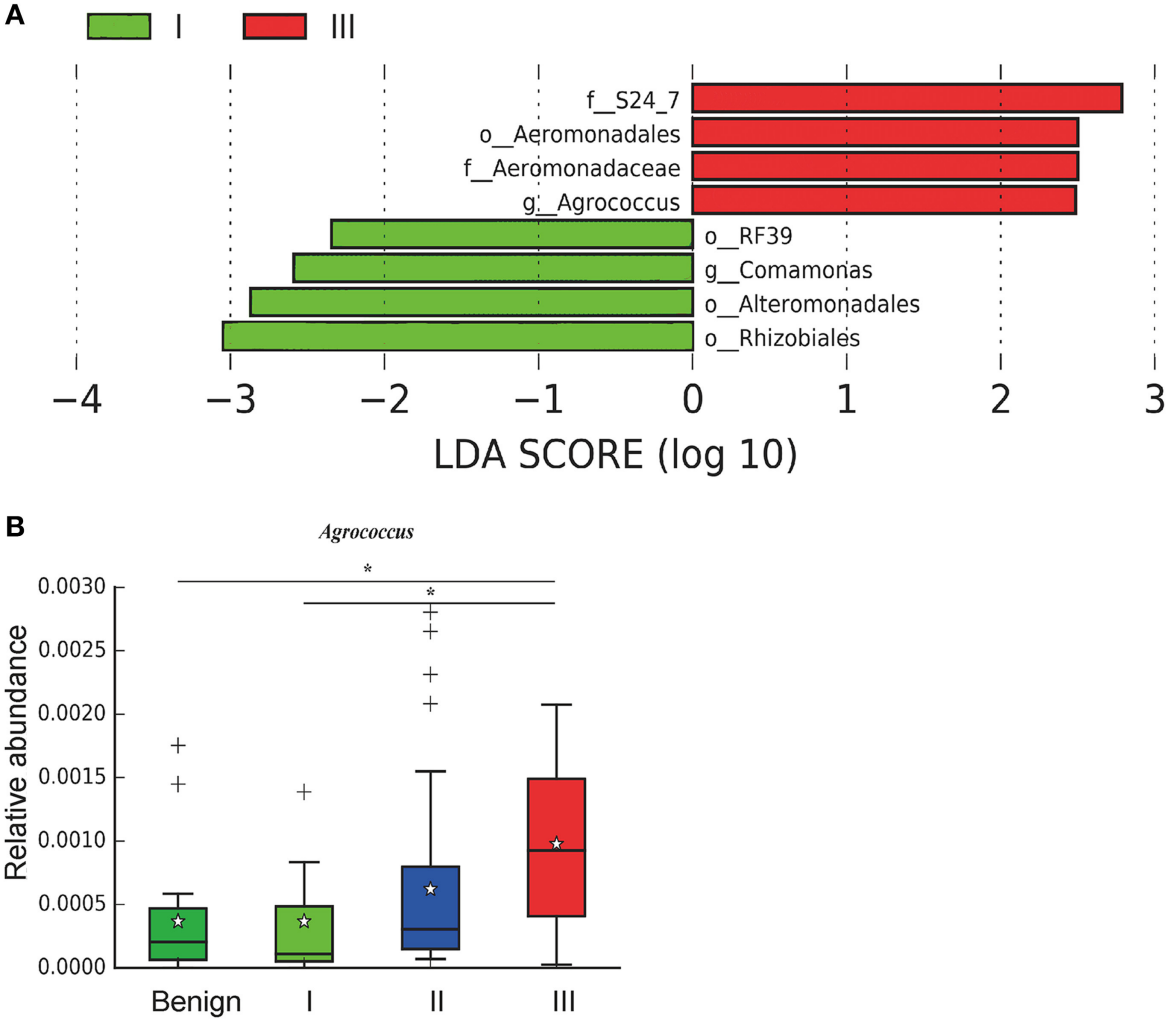
LefSe identified the most differential microbiota in benign, grade I, II, and III. **(A)** Histogram of the LDA scores computed for different abundant in benign, grade I, II, and III. Grade I is indicated with a negative LDA score, and malignant have a positive score. The LDA score indicates the effect size and ranking of each differentially abundant taxon. **(B)** Boxplot compares the relative abundance of *Agrococcus* between different grades of breast tumor tissue (*P* = 0.021, Kruskal-Wilcox's *H*-test). ^*^ indicates that grade I and grade III are significantly different (*P* = 0.049, Welch's *t*-test), benign and grade III are significantly different (*P* = 0.011, Welch's *t*-test).

**Figure 8:**
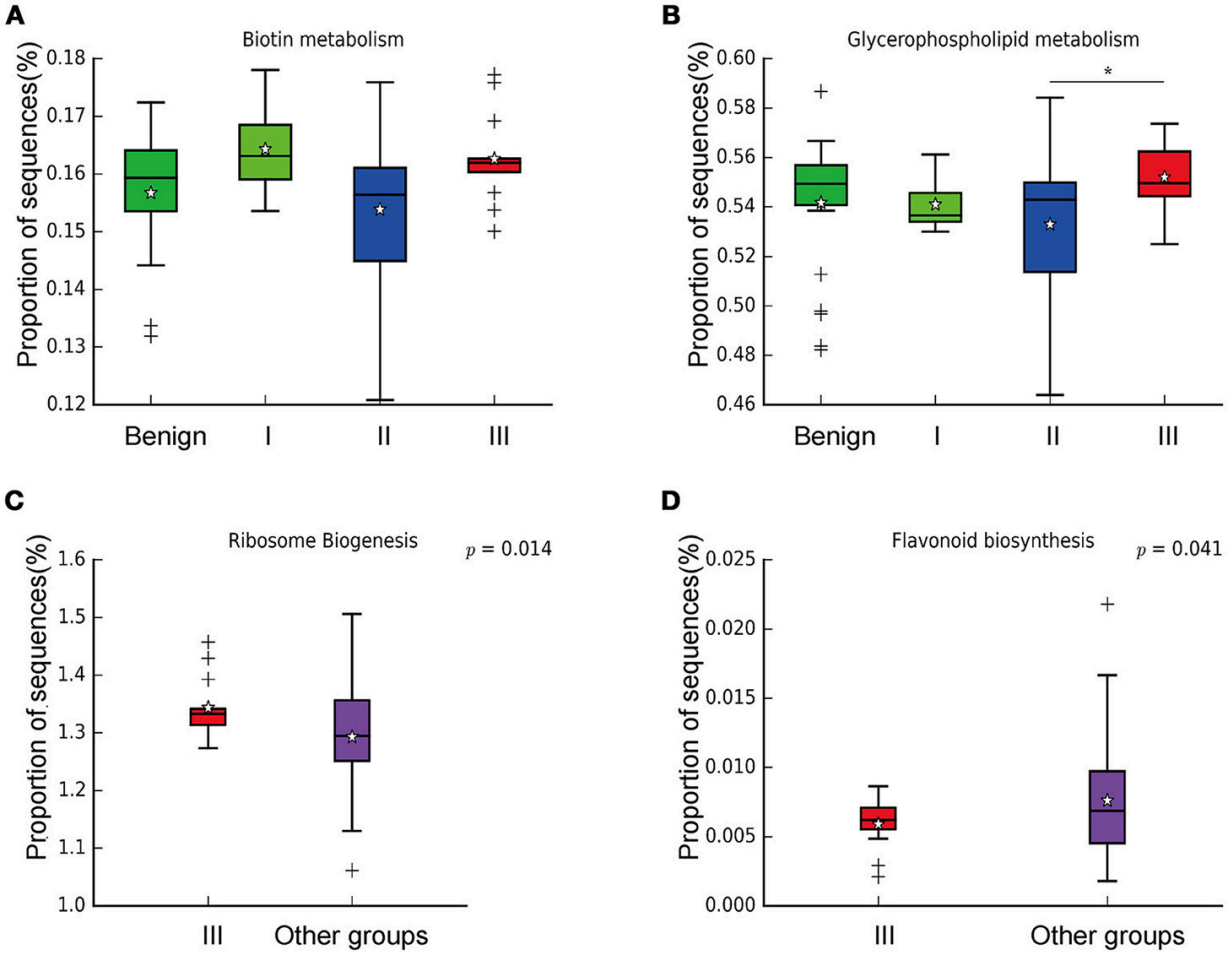
Boxplot showing the significantly different KEGG pathways in benign, grade I, II, and III. **(A)** Biotin metabolism; **(B)** Glycerophospholipid metabolism; ^*^ indicates that grade III is significantly different with grade II (*P* = 0.00264, Welch's *t*-test); **(C)** Ribosome biogenesis; **(D)** Flavonoid biosynthesis.

In order to reveal the differences of microbiomes in different histological grades of malignant tissue, we stratified samples and further analyzed 56 malignant breast tissue samples. According to Nottingham Histologic Score system (the Elston-Ellis modification of Scarff-Bloom-Richardson grading system), patients were stratified into grade I (7 cases), grade II (36 cases), grade III (13 cases). Although there is no significant difference in Shannon index among grade I, II, and III (Figure 5A), the grade III tissue showed higher alpha diversity than grade I and II. We used weighted and unweighted PCoA analysis to explore beta diversity in all three grades, but there were no clear differences (Figures 5B,C). Although the overall microbial composition for different histological grade tissue is similar (Figures 6A,B), the relative abundance of family Bacteroidaceae decreased with increasing malignancy (grade I 10.4% vs. grade II 7.2% vs. grade III 6.9%). LEfSe analysis was used to further discover different compositions of microbiota and identify significant biomarkers (Figure S1). We found that families Aeromonadaceae and S24_7 were enriched in grade III, and order RF39 was enriched in grade I (Figure 7A). The relative abundance of genus *Agrococcus* increased with increasing malignancy (Figure 7B). We also analyzed the effect of several biological/confounding factors on the discovered biomarkers (Table S3). Only 3 of 8 biomarkers were affected by age, menopausal status or ER (*p* < 0.05). PICRUSt was used to infer the KEGG pathways among three histological grades. Biotin metabolism and glycerophospholipid metabolism had significant differences in all three grades (Figures 8A,B). Ribosome biogenesis increased in grade III tissue compared with grade I and II (Figure 8C). Flavonoid biosynthesis was significantly decreased in grade III (Figure 8D).

## Discussion

We collected human breast tissue with aseptic percutaneous needle biopsy and assessed microbiomes with 16S rRNA gene amplicon sequencing. There were three major findings from our study. Previous studies have confirmed the existence of unique microbiota in human breast tissue. We compared microbiome profiles in the breast tissues of patients with benign and malignant disease and identified characteristic microbial biomarkers. Most importantly, we compared the microbiome profiles in breast tissue with different malignant histological grades and identified characteristic microbial biomarkers. This has not been performed in previous studies.

Previous studies in breast cancer tissue microbiome using next-generation sequencing (NGS) include the following studies. Urbaniak et al. (10) used NGS and culture method to analyze breast tissue microbiome taken from 81 breast cancer patients and healthy women from Canada and Ireland. The most abundant phyla in this report were Proteobacteria, followed by Firmicutes, Actinobacteria and Bacteroides. This is similar to our findings at the phyla level. They also found a geographical difference between breast tissue microbiomes of Canadian and Irish subjects. Another study compared the differences in the microbial composition of breast tissue microenvironment in patients with benign vs. malignant disease. They found that malignancy correlated with enrichment in taxa of lower abundance including the genus *Fusobacterium, Atopobium, Gluconacetobacter, Hydrogenophaga*, and *Lactobacillus* (15). However, in our study, genus *Propionicimonas* and five families Micrococcaceae, Caulobacteraceae, Rhodobacteraceae, Nocardioidaceae and Methylobacteriaceae were abundant in malignant disease compared to benign disease. We sought to determine whether there were any common or different bacterial biomarkers/signatures between different study populations, by comparing our data to previous studies that were related to ours (Table S4). Methylobacteriaceae family was found to be the only common biomarker/signature in the tissue microbiomes between tumor/malignant and normal/benign. There are several possible reasons for the inconsistence between our study and others. Firstly, our study used Illumina HiSeq sequencing system to sequence the V1–V2 region of 16S rRNA gene, whereas others used Illumina MiSeq sequencing platform to sequence the V4 region of 16S rRNA gene (9, 11) or Illumina MiSeq sequencing platform to sequence the V3–V5 region of 16S rRNA gene (15). It has been recognized that microbiota compositional data could differ depending on the primers and sequencing platform that were used (22), which is also a potential limitation of this type of research and needs to be solved in future studies. Secondly, our survey characterized the breast microbiomes in Chinese subjects, whereas previous studies exhibited the microbiomes of other ethnicities. It has been demonstrated that eating habits, living environments and metabolic levels in different races can influence the characteristics of the gut and oral microbiome (23, 24). Taken together, these studies indicated that complex microbiota did exist in aseptically collected human breast tissue samples, whereas unique microbiome profiles and biomarkers were discovered in malignant disease samples from different geographical and ethnic cohorts. Our survey includes different microbiomes in benign and malignant disease in Chinese subjects and found that specific bacteria were abundant in malignant disease. The genus *Propionicimonas* which belongs to the family Nocardioidaceae has been reported to be associated with other malignancies such as prostate cancer (25). Xuan et al. identified variation of genus *Methylbacterium* between normal and tumor breast tissue (9). We also detected abundant Methylobacteriaceae, Micrococcaceae and Caulobacteraceae in malignant tumor tissue. Micrococcaceae is associated with bacteremia (26) and ovarian cancer (27). Caulobacteraceae is the common bacteria in urinary tract infections (25).

We further surveyed the potential effects of these bacteria in tumor microenvironment based on KEGG pathways analysis. Thirty one pathways were identified significantly different between benign and malignant disease states. In patients with malignant disease, the pathway nucleotide excision repair was downregulated as compared to that in the benign disease. This pathway has been reported to protect cells and resist cancer (28). The biosynthesis of serine and glycine provides the ability to provide protein for cancer cell growth and is a necessary precursor for nucleic acid and lipid synthesis (29), and they are also important metabolites for cancer cells. Some studies report that metabolic enzymes of serine and glycine biosynthesis are upregulated in cancer (30). We also found that glycine, serine and threonine metabolism pathways were upregulated in malignant disease. This finding provides a new avenue for potential dietary intervention in breast cancer. The pathway drug metabolism—cytochrome P450 and metabolism of xenobiotics by cytochrome P450 were upregulated and drug metabolism for other enzymes was downregulated in malignant disease. This observation has significance in guiding clinical treatment of cancer patients. Pathways in cancer were significantly higher in malignant disease, which indicated that microbes in the tumor microenvironment might be involved in breast cancer progression, which again requires subsequent in-depth studies.

We further examined differences in microbiome of different histological grades of malignant breast tissue. The relative abundance of genus *Agrococcus* (family Microbacteriaceae) increased in malignancy. This bacterium has not been reported in previous studies, which could be a unique and new biomarker for breast cancer. Glycerophospholipid metabolism and ribosome biogenesis pathways were upregulated in grade III tumor compared to grade I and II. Glycerophospholipid metabolism is associated with breast cancer, and in invasive tumors, membrane phospholipid levels are higher (31). This indicates that microbes in the tumor microenvironment could enhance the malignancy of tumors. Ribosome biogenesis is not just excessively activated in early stages of tumor progression, but also in relation to the acquisition of invasive phenotypes, which have also been confirmed in breast cancer (32). Flavonoids have anti-cancer properties and epidemiological studies have shown that a rich flavonoid diet is associated with a reduced risk of breast cancer (33, 34). Our results revealed that flavonoid biosynthesis was significantly lower in grade III compared to grade I and II.

A limitation of our study is the small sample size. Although we calculated the effects of several biologic factors on the biomarkers discovered, the microbiota was still affected by some factors (age and menopausal status). We could not remove other factors influencing microbiota in breast tissue, because the detailed clinical information for all breast cancer patients was not available. For further studies, a larger sample size is needed to identify the influence of microbiota in malignant breast cancer tissue. Another potential limitation could be that KEGG pathways were only predicted by using PICRUSt analysis. PICRUSt can restore the functional information of samples based on the 16S rRNA gene sequencing data (20) and the prediction results is almost consistent (35). The results of function analysis in our study, while biologically plausible, will need to be directly confirmed in future studies by whole-genome methods, such as whole community shotgun sequencing and RNA-seq. Compared with 16S rRNA gene sequence that was used in our study, shotgun metagenomics could offer a more reliable assessment based on functional profiling of the microbiome.

In conclusion, we confirmed the presence of specific microbiota in breast tumor tissue using NGS. We also explored the differences in microbiome profiles in breast tissues between benign and malignant diseases. Most importantly, we compared the microbiomes and their functions in different histological grades of malignant tissue. Taken together, our study can illustrate microbiota in different histological grades of malignant tissue. In addition, the correlation between microbiota and tumor states or histological grade implies that the microbiota in breast tissue might be used in monitoring disease progression in breast cancer. It is still uncertain whether the differences in these microbial communities will promote carcinogenesis, and whether it is due to the increase or the reduction of certain bacteria in the production of cancer cells. Based on our preliminary findings, further investigation of the potential role of tissue microbiome in the development of breast cancer is warrantied. We need to explore how bacteria can affect breast cancer progression, to prevent and treat breast cancer cases. Metagenomics study of the microbiome profile in breast tissue are needed when NGS technology is more advanced, to analyze trace amounts of bacterial DNA in human breast samples using needle biopsy.

## Data availability

All sequencing data associated with this study were uploaded to the GenBank Sequence Read Archive (accession number: SRP154661). The webpage of the SRA database is https://www.ncbi.nlm.nih.gov/sra/.

## Ethics statement

This study was carried out in accordance with the recommendations of Human Specimen Study guidelines of the Institutional Review Board of Qianfoshan Hospital Affiliated to Shandong University with written informed consent from all subjects. All subjects gave written informed consent in accordance with the Declaration of Helsinki. The protocol was approved by the Institutional Review Board of Qianfoshan Hospital Affiliated to Shandong University.

## Author contributions

LZ and QM designed the study. SM, BC, JY, JW, and DZ performed the measurements and data analysis. LZ, JY, and BC wrote the manuscript. All authors have read and critically revised the manuscript.

### Conflict of interest statement

The authors declare that the research was conducted in the absence of any commercial or financial relationships that could be construed as a potential conflict of interest.
