# Supplementary material for: Study of Microbiomes in Aseptically Collected Samples of Human Breast Tissue Using Needle Biopsy and the Potential Role of in situ Tissue Microbiomes for Promoting Malignancy
Source: Front Oncol. 2018 Aug 17;8:318. doi: 10.3389/fonc.2018.00318 (PMC6107834; doi:10.3389/fonc.2018.00318)
Supplement: Supplementary file 1 [file Table_1.DOCX]

**A**


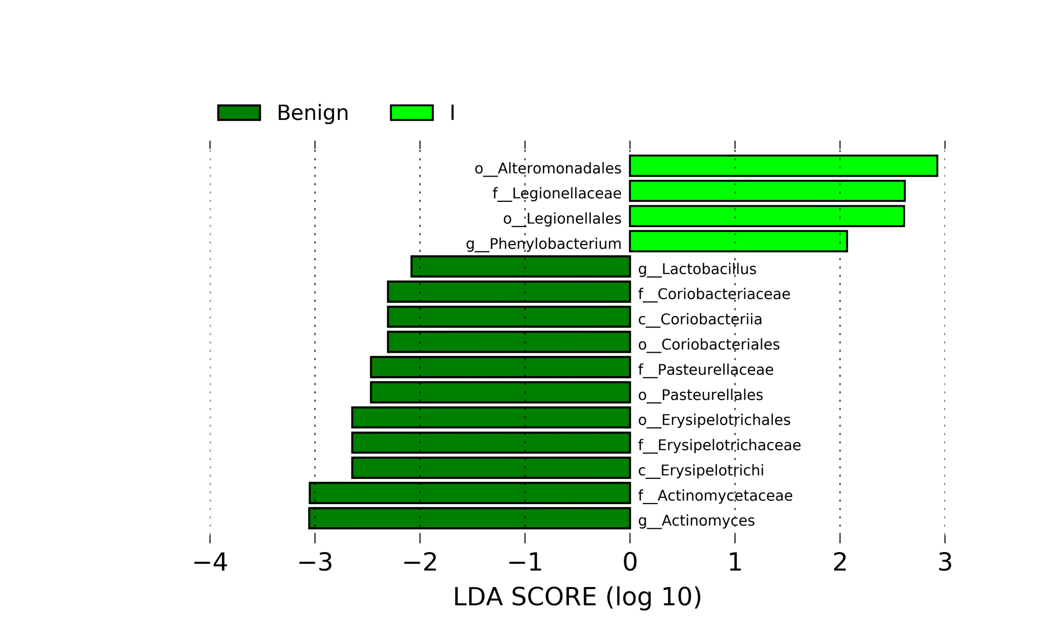


**B**


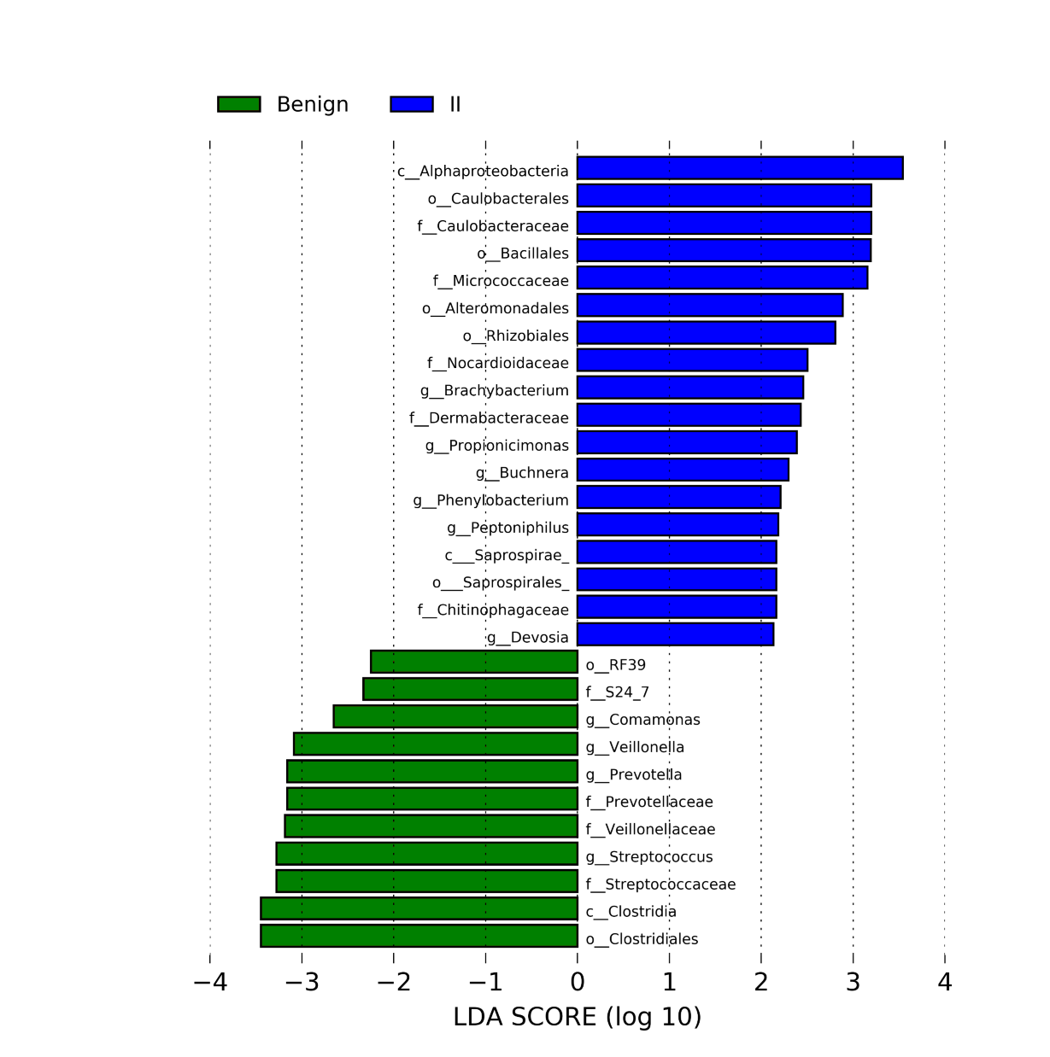


**C**


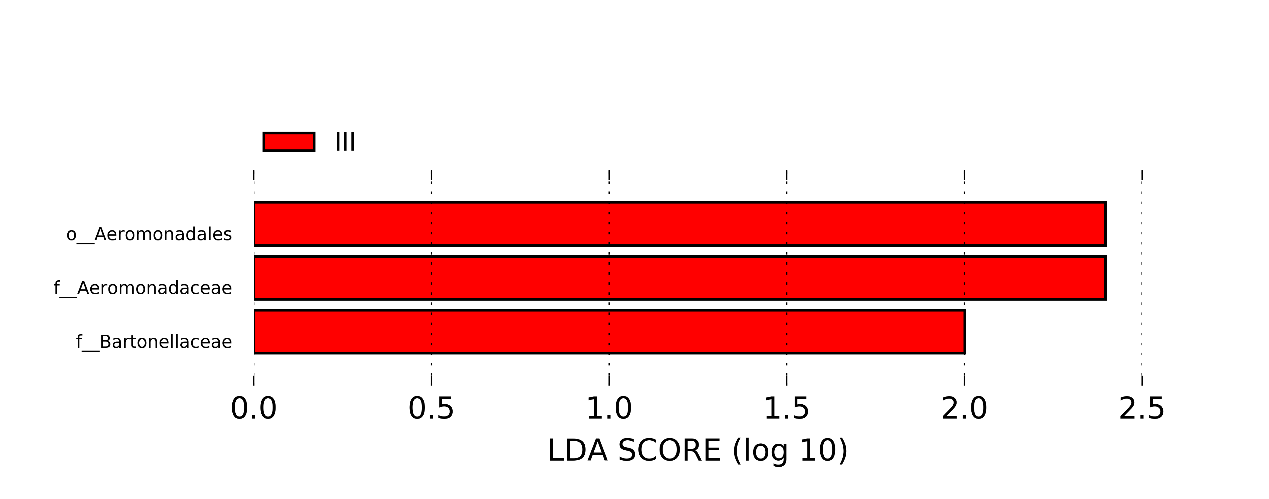


**D**

**
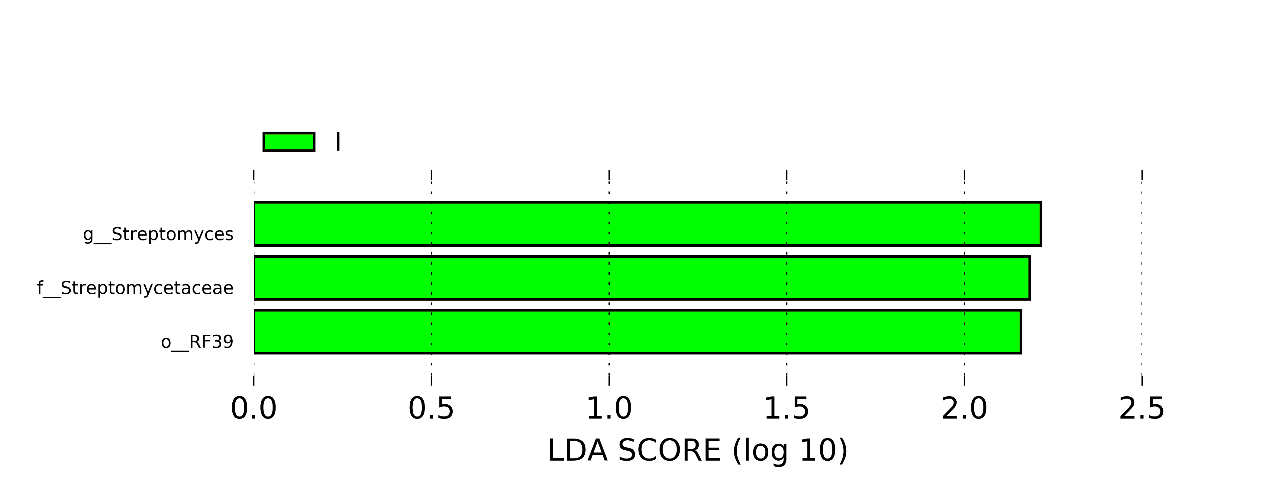
**

**E**


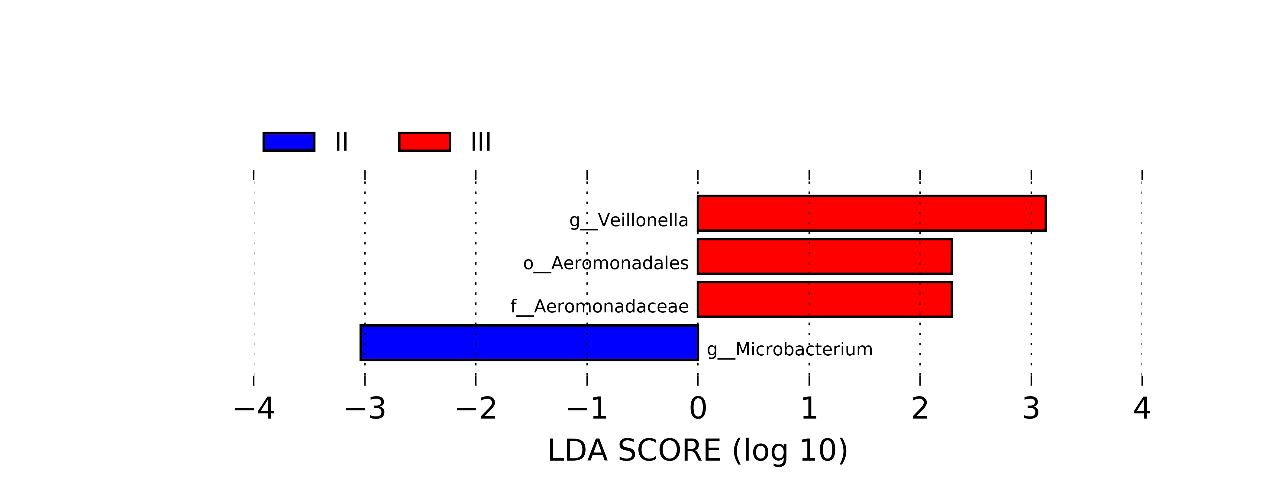


**F**


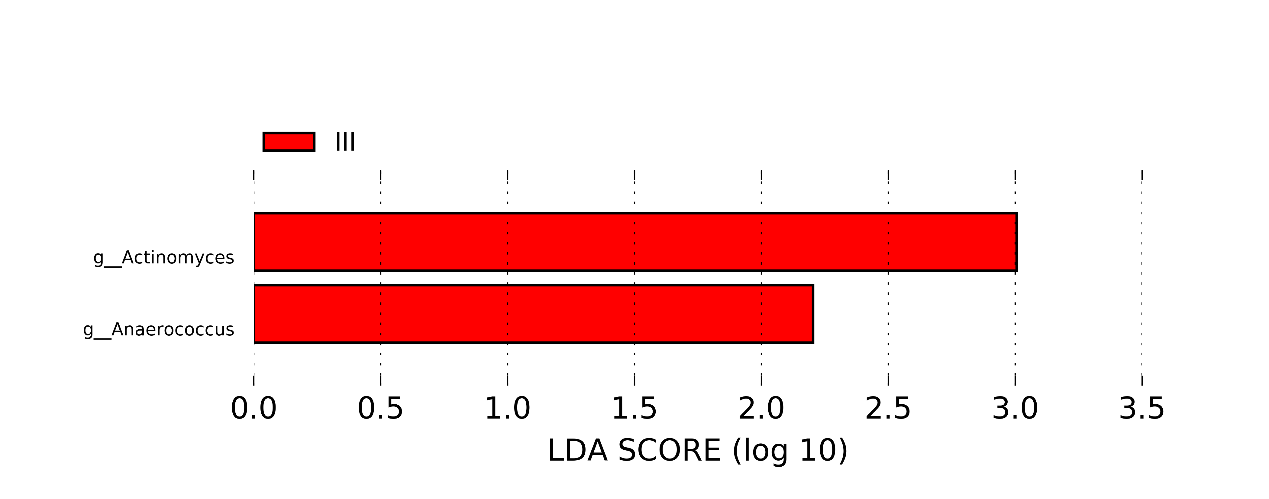


**Figure S1 | LEfSe analysis between benign, Grade I, II and III**

**(A)** Benign vs Grade I. **(B)** Benign vs Grade II. **(C)** Benign vs Grade III. **(D)** Grade I vs Grade II. **(E)** Grade II vs Grade III. **(F)** Grade I vs Grade III.

**Table S1 | Patient, Tumor and Sample Collection Characteristics**

| Sample | Age | Gender | Type | Grade | Type of tumor | Menopausal status | ER | PR | CerB-2 |
| --- | --- | --- | --- | --- | --- | --- | --- | --- | --- |
| B3 | 58 | Female | Benign | Benign | phyllode tumor of the breast | Post-menopause | NA | NA | NA |
| B15 | 46 | Female | Benign | Benign | fibroadenoma of breast | Pre-menopause | NA | NA | NA |
| B17 | 45 | Female | Benign | Benign | fibroadenoma of breast | Pre-menopause | NA | NA | NA |
| B18 | 49 | Female | Benign | Benign | intraductal papilloma | Pre-menopause | NA | NA | NA |
| B20 | 39 | Female | Benign | Benign | benign phyllode tumor of the breast | Pre-menopause | NA | NA | NA |
| B33 | 32 | Female | Benign | Benign | benign phyllode tumor of the breast | Pre-menopause | NA | NA | NA |
| B37 | 52 | Female | Benign | Benign | atypical hyperplasia | Pre-menopause | NA | NA | NA |
| B38 | 49 | Female | Benign | Benign | atypical hyperplasia | Pre-menopause | NA | NA | NA |
| B39 | 49 | Female | Benign | Benign | atypical hyperplasia | Pre-menopause | NA | NA | NA |
| B49 | 54 | Female | Benign | Benign | intraductal papilloma | Post-menopause | NA | NA | NA |
| B69 | 46 | Female | Benign | Benign | atypical hyperplasia | Pre-menopause | NA | NA | NA |
| B73 | 51 | Female | Benign | Benign | atypical hyperplasia | Pre-menopause | NA | NA | NA |
| B81 | 38 | Female | Benign | Benign | atypical hyperplasia | Pre-menopause | NA | NA | NA |
| B86 | 49 | Female | Benign | Benign | atypical hyperplasia | Post-menopause | NA | NA | NA |
| B87 | 60 | Female | Benign | Benign | atypical hyperplasia | Post-menopause | NA | NA | NA |
| B91 | 42 | Female | Benign | Benign | lobular carcinoma in situ | Pre-menopause | NA | NA | NA |
| B97 | 50 | Female | Benign | Benign | intraductal papilloma | Peri-menopause | NA | NA | NA |
| B103 | 46 | Female | Benign | Benign | fibroadenoma of breast | Pre-menopause | NA | NA | NA |
| B106 | 49 | Female | Benign | Benign | atypical hyperplasia | Pre-menopause | NA | NA | NA |
| B107 | 35 | Female | Benign | Benign | atypical hyperplasia | Pre-menopause | NA | NA | NA |
| B115 | 36 | Female | Benign | Benign | benign phyllode tumor of the breast | Pre-menopause | NA | NA | NA |
| B491 | 54 | Female | Benign | Benign | intraductal papilloma | Post-menopause | NA | NA | NA |
| B2 | 72 | Female | Malignant | I | invasive ductal carcinoma of breast | Post-menopause | + | - | + |
| B4 | 69 | Female | Malignant | Malignant | mucinous carcinama of breast | Post-menopause | + | + | - |
| B5 | 63 | Female | Malignant | II | invasive ductal carcinoma of breast | Post-menopause | + | - | + |
| B6 | 47 | Female | Malignant | III | invasive ductal carcinoma of breast | Pre-menopause | - | - | - |
| B7 | 48 | Female | Malignant | II | invasive ductal carcinoma of breast | Pre-menopause | + | + | + |
| B8 | 45 | Female | Malignant | II | invasive ductal carcinoma of breast | Pre-menopause | - | - | + |
| B9 | 50 | Female | Malignant | II | invasive ductal carcinoma of breast | Post-menopause | + | + | + |
| B10 | 45 | Female | Malignant | II | invasive ductal carcinoma of breast | Pre-menopause | + | + | + |
| B11 | 56 | Female | Malignant | Malignant | ductal carcinoma in situ | Post-menopause | - | - | + |
| B12 | 58 | Female | Malignant | II | invasive ductal carcinoma of breast | Post-menopause | + | + | + |
| B13 | 53 | Female | Malignant | II | invasive ductal carcinoma of breast | Post-menopause | - | - | + |
| B14 | 61 | Female | Malignant | Malignant | invasive ductal carcinoma of breast | Post-menopause | + | - | + |
| B16 | 45 | Female | Malignant | II | invasive ductal carcinoma of breast | Post-menopause | + | + | + |
| B19 | 59 | Female | Malignant | II | invasive ductal carcinoma of breast | Post-menopause | + | + | + |
| B22 | 75 | Female | Malignant | II | invasive ductal carcinoma of breast | Post-menopause | - | - | + |
| B23 | 59 | Female | Malignant | II | invasive ductal carcinoma of breast | Post-menopause | + | + | + |
| B24 | 62 | Female | Malignant | II | invasive ductal carcinoma of breast | Post-menopause | - | - | + |
| B25 | 52 | Female | Malignant | II | invasive ductal carcinoma of breast | Post-menopause | + | + | + |
| B26 | 60 | Female | Malignant | II | invasive ductal carcinoma of breast | Post-menopause | + | + | + |
| B27 | 56 | Female | Malignant | Malignant | invasive ductal carcinoma of breast | Post-menopause | - | - | - |
| B31 | 61 | Female | Malignant | II | invasive ductal carcinoma of breast | Post-menopause | + | + | - |
| B32 | 61 | Female | Malignant | Malignant | ductal carcinoma in situ | Post-menopause | + | + | + |
| B34 | 60 | Female | Malignant | Malignant | invasive ductal carcinoma of breast | Post-menopause | + | + | + |
| B35 | 55 | Female | Malignant | Malignant | invasive ductal carcinoma of breast | Post-menopause | + | + | + |
| B36 | 41 | Female | Malignant | II | invasive ductal carcinoma of breast | Pre-menopause | + | - | - |
| B43 | 64 | Female | Malignant | III | invasive ductal carcinoma of breast | Post-menopause | - | - | + |
| B44 | 44 | Female | Malignant | Malignant | osteosarcoma of breast | Pre-menopause | + | + | - |
| B45 | 43 | Female | Malignant | II | invasive ductal carcinoma of breast | Pre-menopause | + | + | + |
| B46 | 29 | Female | Malignant | II | invasive ductal carcinoma of breast | Pre-menopause | + | - | + |
| B47 | 41 | Female | Malignant | II | invasive ductal carcinoma of breast | Pre-menopause | + | + | + |
| B50 | 50 | Female | Malignant | II | invasive ductal carcinoma of breast | Post-menopause | + | + | + |
| B51 | 63 | Female | Malignant | III | invasive ductal carcinoma of breast | Post-menopause | - | - | + |
| B53 | 44 | Female | Malignant | III | invasive ductal carcinoma of breast | Pre-menopause | - | - | + |
| B55 | 60 | Female | Malignant | Malignant | ductal carcinoma in situ | Post-menopause | - | - | + |
| B56 | 42 | Female | Malignant | II | invasive ductal carcinoma of breast | Pre-menopause | + | + | + |
| B58 | 61 | Female | Malignant | I | invasive ductal carcinoma of breast | Post-menopause | + | + | + |
| B59 | 52 | Female | Malignant | II | invasive ductal carcinoma of breast | Pre-menopause | - | - | + |
| B60 | 52 | Female | Malignant | II | invasive ductal carcinoma of breast | Post-menopause | + | + | + |
| B61 | 52 | Female | Malignant | II | invasive ductal carcinoma of breast | Peri-menopause | + | + | + |
| B62 | 33 | Female | Malignant | III | invasive ductal carcinoma of breast | Pre-menopause | - | - | - |
| B63 | 50 | Female | Malignant | II | invasive ductal carcinoma of breast | Post-menopause | + | + | - |
| B64 | 69 | Female | Malignant | II | invasive ductal carcinoma of breast | Post-menopause | + | + | + |
| B65 | 55 | Female | Malignant | III | invasive ductal carcinoma of breast | Post-menopause | + | + | + |
| B66 | 66 | Female | Malignant | II | invasive ductal carcinoma of breast | Post-menopause | + | + | + |
| B71 | 52 | Female | Malignant | I | invasive ductal carcinoma of breast | Pre-menopause | + | + | + |
| B74 | 70 | Female | Malignant | II | invasive ductal carcinoma of breast | Post-menopause | + | + | + |
| B75 | 60 | Female | Malignant | Malignant | mucinous carcinama of breast | Post-menopause | + | + | - |
| B76 | 63 | Female | Malignant | Malignant | diffuse large B cell lymphoma | Post-menopause | - | - | - |
| B77 | 71 | Female | Malignant | I | invasive ductal carcinoma of breast | Post-menopause | + | + | - |
| B80 | 36 | Female | Malignant | Malignant | medullary carcinoma of breast | Pre-menopause | - | - | - |
| B82 | 65 | Female | Malignant | II | invasive ductal carcinoma of breast | Post-menopause | + | + | + |
| B85 | 77 | Female | Malignant | II | invasive ductal carcinoma of breast | Post-menopause | + | + | + |
| B88 | 43 | Female | Malignant | III | invasive ductal carcinoma of breast | Pre-menopause | - | - | - |
| B89 | 50 | Female | Malignant | III | invasive ductal carcinoma of breast | Peri-menopause | - | - | - |
| B90 | 53 | Female | Malignant | II | invasive ductal carcinoma of breast | Post-menopause | + | + | + |
| B93 | 48 | Female | Malignant | II | invasive ductal carcinoma of breast | Pre-menopause | + | + | + |
| B95 | 50 | Female | Malignant | III | invasive ductal carcinoma of breast | Pre-menopause | - | - | + |
| B96 | 38 | Female | Malignant | Malignant | mixed mucinous breast carcinoma | Pre-menopause | + | + | + |
| B98 | 60 | Female | Malignant | I | invasive ductal carcinoma of breast | Post-menopause | + | + | + |
| B99 | 33 | Female | Malignant | Malignant | ductal carcinoma in situ | Pre-menopause | + | + | + |
| B100 | 47 | Female | Malignant | I | ductal carcinoma in situ | Pre-menopause | - | - | - |
| B101 | 45 | Female | Malignant | I | ductal carcinoma in situ | Pre-menopause | - | - | - |
| B104 | 34 | Female | Malignant | III | invasive ductal carcinoma of breast | Pre-menopause | - | - | + |
| B109 | 40 | Female | Malignant | II | invasive ductal carcinoma of breast | Pre-menopause | + | + | + |
| B112 | 74 | Female | Malignant | II | invasive ductal carcinoma of breast | Post-menopause | + | + | + |
| B114 | 40 | Female | Malignant | Malignant | invasive ductal carcinoma of breast | Pre-menopause | + | + | - |
| B116 | 60 | Female | Malignant | II | invasive ductal carcinoma of breast | Post-menopause | - | - | + |
| B117 | 50 | Female | Malignant | II | invasive ductal carcinoma of breast | Post-menopause | + | + | + |
| B118 | 55 | Female | Malignant | III | invasive ductal carcinoma of breast | Post-menopause | - | - | + |
| B120 | 76 | Female | Malignant | III | invasive ductal carcinoma of breast | Post-menopause | - | - | - |
| B123 | 66 | Female | Malignant | III | invasive ductal carcinoma of breast | Post-menopause | - | - | - |
| B251 | 52 | Female | Malignant | Malignant | invasive ductal carcinoma of breast | Post-menopause | + | + | + |

**Table S2 |** **Effect of several biologic factors on the discovered biomarkers for Benign *vs.* Malignant.**

| Biomarker | Benign | |  | Malignant | | | | |
| --- | --- | --- | --- | --- | --- | --- | --- | --- |
|  | Age | Menopausal status |  | Age | Menopausal status | ER | PR | CerB02 |
| p__Proteobacteria | 0.1558459 | 0.203581758 |  | 0.909371 | 0.98777 | 0.598855 | 0.808709 | 0.226805328 |
| c__Acidobacteria-6 | 0.8675425 | 0.190492437 |  | 0.054037 | 0.14693 | 0.955851 | 0.9987 | **0.044003973** |
| c__Clostridia | 0.3468569 | **0.007260157** |  | 0.954402 | 0.66757 | 0.562093 | 0.978973 | 0.709848186 |
| c__Alphaproteobacteria | **0.011527** | **0.031920579** |  | 0.989513 | 0.80354 | 0.440153 | 0.502809 | 0.541243389 |
| o__iii1-15 | 0.8675425 | 0.190492437 |  | 0.054037 | 0.14693 | 0.955851 | 0.9987 | **0.044003973** |
| o__Bacillales | 0.1930981 | **0.011786029** |  | 0.997334 | 0.48235 | 0.236599 | 0.124222 | 0.135351729 |
| o__Clostridiales | 0.3468569 | **0.007260157** |  | 0.954402 | 0.66757 | 0.562093 | 0.978973 | 0.709848186 |
| o__Caulobacterales | **0.0062594** | 0.108741578 |  | 0.745412 | 0.86406 | 0.5615 | 0.455634 | 0.600187551 |
| o__Rhizobiales | 0.685409 | 0.385094319 |  | 0.395333 | 0.99555 | 0.234896 | 0.514322 | 0.945345046 |
| o__Rhodobacterales | 0.2465923 | 0.455518828 |  | 0.999997 | 0.88875 | 0.195143 | 0.118508 | 0.994163787 |
| o__Alteromonadales | **0.0086151** | 0.418797412 |  | **0.045015** | 0.58153 | 0.654304 | 0.412858 | 0.497641051 |
| f__Micrococcaceae | 0.7306374 | 0.055878777 |  | 0.133704 | 0.26849 | 0.24317 | 0.717329 | 0.18103593 |
| f__Nocardioidaceae | 0.9315933 | 0.156322217 |  | 0.242337 | 0.70488 | 0.207036 | 0.121655 | 0.374211449 |
| f__Prevotellaceae | 0.2540468 | **0.013607864** |  | 0.945823 | 0.88369 | 0.742168 | 0.754058 | 0.844318908 |
| f__Veillonellaceae | 0.6501206 | **0.007503729** |  | 0.922929 | 0.65274 | 0.329952 | 0.614158 | 0.842079738 |
| f__Caulobacteraceae | **0.0062594** | 0.108741578 |  | 0.745412 | 0.86406 | 0.5615 | 0.455634 | 0.600187551 |
| f__Methylobacteriaceae | 0.7165153 | 0.306543827 |  | 0.411151 | 0.96704 | 0.706679 | 0.800563 | 0.051552541 |
| f__Rhodobacteraceae | 0.2465923 | 0.455518828 |  | 0.999997 | 0.88875 | 0.195143 | 0.118508 | 0.994163787 |
| g__Propionicimonas | 0.8057839 | 0.513535205 |  | 0.214382 | 0.62621 | 0.339616 | 0.252202 | 0.400591803 |
| g__Prevotella | 0.2540468 | **0.013607864** |  | 0.945823 | 0.88369 | 0.742168 | 0.754058 | 0.844318908 |
| g__Veillonella | 0.7153731 | **0.018537286** |  | 0.928834 | 0.82973 | 0.222663 | 0.386393 | 0.968063485 |

**Table S3 | Effect of several biologic factors on the discovered biomarkers for different grade.**

| Biomarker | I vs II vs III | | | | |
| --- | --- | --- | --- | --- | --- |
|  | Age | Menopausal status | ER | PR | CerB02 |
| g__Agrococcus | **0.0096033** | **0.018887409** | 0.527736 | 0.346588 | 0.55538 |
| f__S247 | 0.8884088 | 0.654956296 | 0.499322 | 0.302171 | 0.50791 |
| o__Ardenscatenales | 0.1679797 | 0.723548268 | 0.602155 | 0.484678 | 0.63808 |
| o__Rhizobiales | 0.9842048 | 0.643415791 | 0.066586 | 0.199395 | 0.85129 |
| g__Comamonas | 0.1761129 | 0.343928384 | 0.166298 | 0.37839 | 0.12166 |
| f__Aeromonadaceae | 0.75028 | 0.981992389 | 0.426592 | 0.498099 | 0.43237 |
| o__Alteromonadales | 0.171851 | 0.083593594 | 0.317948 | 0.212743 | 0.32435 |
| o__RF39 | 0.2858641 | 0.841965627 | **0.04238** | 0.24559 | 0.25195 |

The effects of each of the two factors, i.e. Age on the validated biomarkers were examined by the one way ANOVA test.

**Table S4 | The biomarker in our study comparison with other studies**

| **Microbiota** | **Change** | **Sample type** | **Research** | **Populations** |
| --- | --- | --- | --- | --- |
| Sphingomonadaceae family | ↓ | Tumor vs Normal | Xuan *et al*., 2016, PLoS One | American |
| *Sphingomonas* genus | ↓ | Tumor vs Normal | Xuan *et al*., 2016, PLoS One | American |
| *Sphingomonas yanoikuyae* | ↓ | Tumor vs Normal | Xuan *et al*., 2016, PLoS One | American |
| **Methylobacteriaceae family** | **↑** | **Tumor vs Normal** | **Xuan *et al*., 2016, PLoS One** | **American** |
| *Methylobacterium* genus | ↑ | Tumor vs Normal | Xuan *et al*., 2016, PLoS One | American |
| *Methylobacterium radiotolerans* | ↑ | Tumor vs Normal | Xuan *et al*., 2016, PLoS One | American |
| Sphingomonadaceae family | ↓ | Tumor vs Normal | Chan *et al*., 2016, Scientific Reports | American |
| *Alistipes* genus | ↑ | Tumor vs Normal | Chan *et al*., 2016, Scientific Reports | American |
| Fusobacteriaceae family | ↑ | Malignant vs Benign | Hieken *et al*., 2016, Scientific Reports | American |
| *Fusobacterium* genus | ↑ | Malignant vs Benign | Hieken *et al*., 2016, Scientific Reports | American |
| Coriobacteriaceae family | ↑ | Malignant vs Benign | Hieken *et al*., 2016, Scientific Reports | American |
| *Atopobium* genus | ↑ | Malignant vs Benign | Hieken *et al*., 2016, Scientific Reports | American |
| *Gluconacetobacter* genus | ↑ | Malignant vs Benign | Hieken *et al*., 2016, Scientific Reports | American |
| Tissierellaceae family | ↑ | Malignant vs Benign | Hieken *et al*., 2016, Scientific Reports | American |
| *Hydrogenophaga* genus | ↑ | Malignant vs Benign | Hieken *et al*., 2016, Scientific Reports | American |
| Lactobacillaceae family | ↑ | Malignant vs Benign | Hieken *et al*., 2016, Scientific Reports | American |
| *Lactobacillus* genus | ↑ | Malignant vs Benign | Hieken *et al*., 2016, Scientific Reports | American |
| Mogibacteriaceae family | ↓ | Malignant vs Benign | Hieken *et al*., 2016, Scientific Reports | American |
| **Methylobacteriaceae family** | **↑** | **Malignant vs Benign** | **Our study** | **Chinese** |
| *Propionicimonas* genus | ↑ | Malignant vs Benign | Our study | Chinese |
| Nocardioidaceae family | ↑ | Malignant vs Benign | Our study | Chinese |
| Rhodobacteraceae family | ↑ | Malignant vs Benign | Our study | Chinese |
| Caulobacteraceae family | ↑ | Malignant vs Benign | Our study | Chinese |
| Micrococcaceae family | ↑ | Malignant vs Benign | Our study | Chinese |
| Veillonellaceae family | ↓ | Malignant vs Benign | Our study | Chinese |
| *Veillonella* genus | ↓ | Malignant vs Benign | Our study | Chinese |
| Prevotellaceae family | ↓ | Malignant vs Benign | Our study | Chinese |
| *Prevotella* genus | ↓ | Malignant vs Benign | Our study | Chinese |
